# Distributed Subnetworks of Depression Defined by Direct Intracranial Neurophysiology

**DOI:** 10.3389/fnhum.2021.746499

**Published:** 2021-10-21

**Authors:** Katherine Wilson Scangos, Ankit N. Khambhati, Patrick M. Daly, Lucy W. Owen, Jeremy R. Manning, Josiah B. Ambrose, Everett Austin, Heather E. Dawes, Andrew D. Krystal, Edward F. Chang

**Affiliations:** ^1^Department of Psychiatry, University of California, San Francisco, San Francisco, CA, United States; ^2^Weill Institute for Neurosciences, University of California, San Francisco, San Francisco, CA, United States; ^3^Department of Neurological Surgery, University of California, San Francisco, San Francisco, CA, United States; ^4^Department of Psychological and Brain Sciences, Dartmouth College, Hanover, NH, United States; ^5^Kaiser Permanente Redwood City Medical Center, Redwood City, CA, United States

**Keywords:** biomarkers, biotypes, depression, ECoG, EEG

## Abstract

Major depressive disorder is a common and disabling disorder with high rates of treatment resistance. Evidence suggests it is characterized by distributed network dysfunction that may be variable across patients, challenging the identification of quantitative biological substrates. We carried out this study to determine whether application of a novel computational approach to a large sample of high spatiotemporal resolution direct neural recordings in humans could unlock the functional organization and coordinated activity patterns of depression networks. This group level analysis of depression networks from heterogenous intracranial recordings was possible due to application of a correlational model-based method for inferring whole-brain neural activity. We then applied a network framework to discover brain dynamics across this model that could classify depression. We found a highly distributed pattern of neural activity and connectivity across cortical and subcortical structures that was present in the majority of depressed subjects. Furthermore, we found that this depression signature consisted of two subnetworks across individuals. The first was characterized by left temporal lobe hypoconnectivity and pathological beta activity. The second was characterized by a hypoactive, but hyperconnected left frontal cortex. These findings have applications toward personalization of therapy.

## Introduction

Major depressive disorder (MDD) is a common, highly disabling and potentially deadly disorder that affects more than 264 million individuals worldwide (G. B. D. Disease Injury Incidence Prevalence Collaborators, 2018). Despite significant neuroscientific advances, the biological substrate of depression remains poorly understood and new approaches that facilitate our understanding are critical. The majority of early studies seeking to characterize depression pathophysiology examined specific brain regions [ex. subgenual anterior cingulate cortex (Kennedy et al., 2001; Botteron et al., 2002; Yoshimura et al., 2010)], cognitive networks [ex. default mode network (Greicius et al., 2007; Bluhm et al., 2009; Grimm et al., 2009; Sheline et al., 2010; Zhu et al., 2012)], or univariate electrophysiological markers [ex. alpha asymmetry (Henriques and Davidson, 1991; Gotlib et al., 1998; Kentgen et al., 2000; Diego et al., 2001; Kemp et al., 2010; Jaworska et al., 2012)]. Yet, there is increasing evidence that depression is characterized by distributed network dysfunction beyond a single brain region or network (Veer et al., 2010; Zeng et al., 2012; Liu et al., 2013).

Recent computational advancements within a network neuroscience framework have enabled researchers to model brain activity with the scope and complexity necessary to understand such distributed processes (Bassett and Sporns, 2017). However, detailed investigations of both the functional organization and coordinated activity patterns of depression networks have been limited by the capabilities of current imaging and electroencephalography (EEG) technologies, both indirect measures of neural activity that require a trade-off between spatial and temporal resolution. Intracranial EEG (iEEG), typically collected in patients with epilepsy for the purpose of seizure localization, has the advantage of high temporal resolution, and provides direct recordings from both cortical and subcortical brain structures. Patients with epilepsy have high rates of co-morbid depression (Hermann et al., 2000; Gilliam et al., 2003; Swinkels et al., 2005; Hermann and Jones, 2006; Fuller-Thomson and Brennenstuhl, 2009; Rai et al., 2012) that shares origin (Schmitz, 2006; Mula and Schmitz, 2009; Vezzani et al., 2011; Gleichgerrcht et al., 2015; Wulsin et al., 2016) and treatment response (Kanner, 2003) characteristics with primary depression. However, owing to heterogenous electrode placement across individuals, previous iEEG studies have been limited to low patient numbers and region-based approaches (Kirkby et al., 2018; Sani et al., 2018; Scangos et al., 2019a).

We hypothesized that we could apply a novel computational approach to a large unique dataset of multi-region, multi-day iEEG recordings in 54 participants to uncover distributed cortico-subcortical networks in depression. To tackle inconsistent network sampling across individuals, we utilized a method called SuperEEG (Owen et al., 2020) that uses the correlational structure of brain activity across the population to create a model of multiregional iEEG activity for each individual despite heterogeneous electrode placement. This model provided a highly detailed representation of brain activity across space and time and allowed us to chart out the inherent organization of the brain into functional networks. Once a generalized map of functional brain network organization was established, we quantified the multi-dimensional nature of corresponding brain dynamics to discover how rhythmic activity riding atop these functional networks differed in depressed and non-depressed individuals (Gu et al., 2018). Because depression has a variable presentation, we further examined how depression-associated circuitry varied across individuals in the depressed group.

We found that depression circuitry was highly distributed across cortical and subcortical structures with global dysfunction in both connectivity and spectral activity. Two unique depression subnetworks present in 89% of depressed subjects were identified. One pattern was marked by decreased connectivity across the occipitotemporal region and dominant beta band activity. The second was characterized by excessive frontal cortical connectivity with decreased activity in the alpha spectral frequency band.

## Materials and Methods

### Patient Characterization

Participants included 54 adults (27 female) aged 20–67 who had medication-refractory epilepsy and were undergoing intracranial mapping with multi-channel iEEG for seizure localization as part of their standard medical care (Supplementary Table 1). Neural data from these participants comprised our full dataset and was utilized to build the whole-brain iEEG model of LFP time-series. Participants were screened for depression following electrode implantation and concurrent with neural recordings using the Patient Health Questionnaire-9 (PHQ-9), a 9-item self-report instrument validated for depression screening (Supplementary Figure 1A; Spitzer et al., 1999, 2000; Kroenke et al., 2001). A score ≥ 10 defined the depressed group (moderate depression) and a score ≤ 5 defined the non-depressed control group generating a sample of 23 depressed subjects (56%) and 18 controls (44%). A cut-off score of 10 was selected to define the depressed group because it is the standard threshold used for screening in clinical practice, was defined by the scale’s developer, and has been used in large-scale validation studies (Kroenke et al., 2001; Arroll et al., 2010; Levis et al., 2019). The remaining 13 patients were used in the first step of the study (*Model building*) but not the second (*Model utilization*). Data comprised a consecutive series of patients recruited from University of California, San Francisco and Kaiser Permanente, Redwood City, California over a 5-year period. This study was approved by the University of California, San Francisco Institutional Review Board with written informed consent provided by all subjects. Patients’ antiepileptic medications (AEDs) were withdrawn as part of standard clinical care. However, to control for possible effects of medication on neural activity in the depressed and control groups we examined the number of patients in each group that were on AEDs associated with depression (Nadkarni and Devinsky, 2005) using a chi squared test.

### Electrode Implantation and Localization

Subdural grid, strip, and depth electrodes (AdTech, Racine, WI, United States; or Integra, Plainsboro, NJ, United States) were implanted using standard neurosurgical techniques. The number of electrodes per subject ranged from 33 to 201 (mean = 120, SD = 37). Subjects underwent pre-operative 3 Tesla brain magnetic resonance imaging (MRI) and post-operative computed tomography (CT) scan to localize electrodes in patient-centered coordinates using an open source python package for preprocessing imaging data for use in iEEG recordings (Hamilton et al., 2017). The steps included warping brain reconstructions to a common Montreal Neurologic Institute (MNI) template and merging electrode locations across subjects. Surface warpings were then generated by projecting pial surfaces of the subject and template brains into a spherical coordinate space and aligning the surfaces in that space. Depth warping was then performed using a combination of volumetric and surface warping (Postelnicu et al., 2009). For visualization, pre-operative T1-weighted MRI scans were pre-registered with the post-operative CT using Statistical Parametric Mapping software SPM12 and pial surface 3D reconstructions were generated using FreeSurfer (Fischl, 2012).

### Data Acquisition and Pre-processing

Data acquisition of iEEG recordings were acquired using the Natus EEG clinical recording system at a sampling rate of 1–2 kHz. Standard iEEG/ECoG pre-processing techniques were conducted in python including application of a 2–250 Hz bandpass filter, notch filters at line noise frequency and harmonics (60, 120, 180, and 240 Hz), down sampling to 512 Hz, and common average referencing to the mean of all channels. The data were acquired across a range of behaviors while the patient was in the epilepsy monitoring unit.

### Overall Approach

**FIGURE 1:**
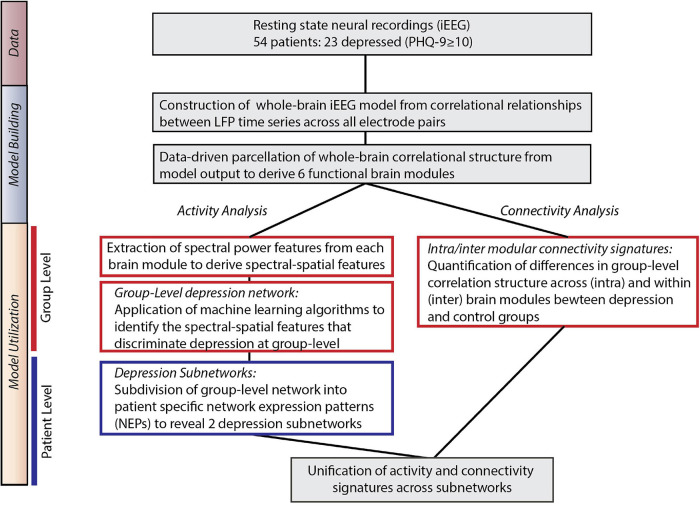
Overall approach. *Model Building:* We utilized direct neural recordings from 54 patients to construct a whole-brain model of iEEG activity based on correlational relationships of neural LFP time series signals across all electrode pairs. We then parcellated this model into functional network modules using graph theory metrics. *Model Utilization:* We used the whole-brain iEEG model to study how brain activity and connectivity measures relate to depression status. We first defined spectral power features across network modules and applied supervised machine learning to identify a group-level network features of depression (*Activity analysis*). In parallel, we identified alterations in functional network connectivity and organization between depressed and control groups (*Connectivity analysis*). Common group-level network features expressed at the individual level were clustered to identify two distinct patterns of altered activity and connectivity.

Our overall approach consisted of two steps – a model building step where we identified large-scale functional networks across iEEG electrodes, and a model utilization step where we related the architecture and intrinsic neural activity of functional networks to depression status (Figure 1).

### Construction of Whole-Brain Intracranial EEG Model

**FIGURE 2:**
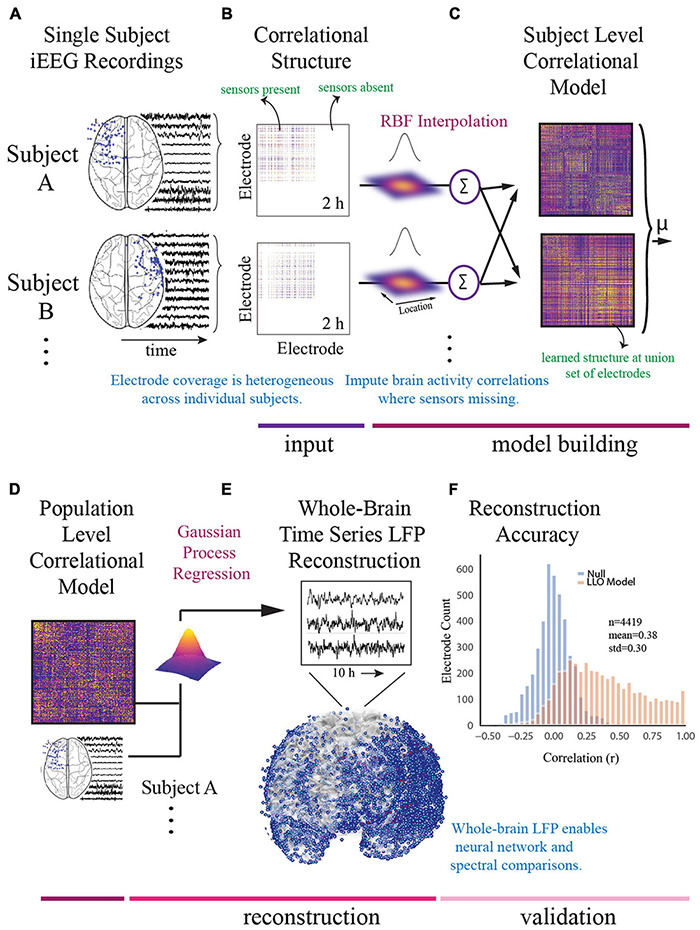
Construction of whole-brain model. **(A)** To generate a multi-subject whole-brain model of iEEG activity, patient’s electrode locations across participants were first represented in a common space [Montreal Neurologic Institute (MNI) space]. Electrode locations and sample recordings for a few example patients are shown. Activity was then randomly sampled in 1 min intervals across daytime hours to obtain a stable representation of brain activity across a 2-h period. **(B)** Individual inter-electrode correlation matrices were constructed for each participant at locations where electrodes were present. **(C)** Subject-level full brain correlational models were then predicted using radial basis function (RBF)-weighted averages to estimate brain activity correlations at locations where sensors were not present. **(D)** Subject-level correlational models were then averaged to generate a population level whole-brain correlational model. **(E)** Local field potential activity for each of the 4,244 electrodes was then reconstructed using Gaussian process regression with the population-level model as a prior and activity where electrodes were present as the marginal likelihood. **(F)** The distribution of the electrode signal reconstruction accuracy across true correlational models (orange) compared to reconstruction accuracy obtained from shuffled correlational models. To obtain this distribution we built models with 53/54 patients, and then applied the model to the held-out patient, holding out each patient in turn. Correlation of the true and reconstructed signals were compared for each held-out electrode. Significance was assessed by averaging the patient level Fisher transformed correlation coefficients and comparing the distribution for the true correlational model and the shuffled correlational model using a *t*-test (*t* = 13.94, *p* = 1.04e^− 25^).

For the model building step, we used a functional connectivity imputation technique, called SuperEEG (Owen et al., 2020) to map continuous iEEG recordings from different patients into a common neural space (Figure 2). This method provided an important advance over previous iEEG studies (Kirkby et al., 2018; Sani et al., 2018; Scangos et al., 2019a) that were limited to region-based analyses conducted in small samples due to heterogeneous electrode placement. To generate this model, pre-processed iEEG signals were chunked into 60 s non-overlapping blocks and filtered for putative epileptiform activity or artifacts using kurtosis, a measure of infrequent extreme peaked deviations (Akbarian and Erfanian, 2018; Owen et al., 2020). We then randomly sampled the 60 s intervals across daytime hours (8am–10pm) and concatenated them into 2-h blocks, each representative of naturalistic activity. We then constructed subject-level whole-brain correlational models. To do so, interelectrode correlation matrices were constructed from activity where sensors were present and learned radial-basis function weighted averages were used to generate correlational information at locations where sensors were not present. The subject-level models were then averaged to generate a population-level model. We then used Gaussian process regression based on the population-level model and individual time series for each subject to reconstruct whole-brain local field potentials for each subject. Evaluation of the SuperEEG algorithm has been performed previously on two large independent iEEG datasets using leave-one-out cross-validation (Owen et al., 2020). Reconstruction accuracy was measured by calculating the correlation between the true and reconstructed signals for each held-out electrode from the held-out patients. By using only other patients’ data to estimate activity for each held-out electrode, volume conductance or other sources of “leakage” were minimized resulting in a conservative estimate of reconstruction accuracy. Using the same approach as Owen et al. (2020), we compared the reconstruction accuracies obtained by the true held-out models (mean *r* = 0.38) to the reconstruction accuracies obtained by shuffled held-out models (mean *r* = 0.00) in which the interelectrode correlations of the SuperEEG model were permuted uniformly to generate activity patterns that would be reconstructed by chance. As we hypothesized, we found that the reconstruction accuracies for the true held-out models were significantly greater than the reconstruction accuracies of the shuffled held-out models (*t* = 13.94, *p* = 1.04e^–25^), suggesting that the SuperEEG model reconstructs activity patterns significantly better than chance.

The SuperEEG algorithm requires extensive computational resources. Therefore, we sought to utilize the minimum required information to obtain the majority of information and enable computational feasibility. Using the 10 h benchmark as the largest feasible model we could build, we compared 2, 4, 6, and 8 h models to the 10 h model and found that the difference in adding additional time beyond 2 h was marginal and could be computed at a fraction of the computational cost. We therefore utilized the 2 h model for further analysis (Supplementary Figure 1B).

### Signal Processing

Standard signal processing techniques were applied to the time-series activity across all reconstructed electrodes. This included continuous wavelet transformation using the Morlet transform wavelet method (6-cycles) (Schiff et al., 1994) performed in 30 s intervals to obtain power spectra in 6 frequency bands (delta = 1–4 Hz, theta = 5–8 Hz, alpha = 9–12 Hz, beta = 13–30 Hz, low gamma (gammaL) = 31–70 Hz, high gamma (gammaH) = 71–150 Hz). Relative power was calculated by dividing the power of each frequency band by the total power across the 6 frequency bands for each electrode. Signals were summarized by taking the mean power across time for each spectral band and were z-scored across patients.

### Electrode Clustering Into Functional Modules

After construction of the full-brain correlational model, we next utilized principles of graph theory to identify data-driven functional networks (modules) across it. Our rationale was that the model had learned statistically correlated fluctuations between iEEG signals, akin to functional connectivity, and that a network-based approach could enhance discovery of depression circuitry over a univariate, single-region approach. We used a well-validated modularity optimization technique known as multiscale community detection, which groups electrodes into non-overlapping modules by their correlational relationships (Newman, 2006; Blondel et al., 2008) and has been used to reveal system-level disruptions in disease states (Alexander-Bloch et al., 2010; Chen et al., 2011; Yu et al., 2011; Bruno et al., 2012; Cao et al., 2014; Sun et al., 2014; Keown et al., 2017) including MDD (He et al., 2018). We conceptualize a network module as a distinct property of connectivity organization, akin to validated atlas parcellations (Cammoun et al., 2012) but specifically designed for functional rather than structural data. Atlases apply boundaries to brain regions based on structural or functional organization derived from coarse-scale neuroimaging and thus, while they provide a useful validation for our data-driven parcellation scheme, there is no reason to assume their boundaries will perfectly align with neural signals at the millimeter scale of iEEG.

Individual functional connectivity models generated in the whole-brain iEEG reconstruction were used as a starting point in this analysis. Using the Louvain algorithm (Blondel et al., 2008), we identified an optimal parcellation of electrodes into discrete functional modules by maximizing a modularity cost function defined by the following relationships,


(1) $$P=\frac{2}{{\left|K\right|}_{F}}KJK^{T}$$

(2) $$Q={\left|{\left(K-\gamma P\right)}^{\circ }G\right|}_{F}$$

where *J* is a ones matrix, ° is the Hadamard product and Gi,j is 0 if node pair (*i*,*j*) are assigned to different modules and 1 if the pair is assigned to the same module, *Q* is modularity, *K* is the connection weights (correlation) between node*i* and *j*, *P* is the Newman-Girvan null model (Newman, 2006) and γ is the weighting of that null model which is tuned to obtain network modules of different sizes. Previous work on module detection (Bassett et al., 2013) demonstrated that tuning this resolution parameter is key to identifying modules at different topological scales of a network. We examined network modularity at values of γ between 0.5 and 2.1. We first assessed the stability of clustering at each value of γ by examining module allegiance (Bassett et al., 2015), calculated by repeating module detection 100 times and evaluating the probability that two electrodes occupied the same module.

Then, in line with previous efforts that have related iEEG network structure to brain parcellations based on anatomy (Betzel et al., 2019), we computed a similarity index (Misic et al., 2016) between the division of electrodes into modules and the division of electrodes into the 234 anatomically distinct brain areas defined by the Lausanne atlas (Cammoun et al., 2012) for the range of resolution parameters (Supplementary Figure 1C). Significance was assessed by a permutation test where the null model was generated by randomly assigning electrodes to each module and calculating the confidence interval of the similarity index generated from 1,000 random permutations and tested at significance level 0.05 for a two-tailed test. Two similarity peaks were identified, with values of γ that generated 6 and 1,855 modules, respectively. The peak with the highest modularity (lowest number of clusters) was selected for further analysis due to our goal of examining the brain at a low level of granularity. This selection enabled subsequent classification of activity across these clusters without overfitting our model. While we report our results based on this most parsimonious match between modules and anatomical structures (γ = 1.19), we verified that the assignment of electrodes into slightly coarser and slightly finer modules (1 < γ < 2.1) did not substantially alter our ability to predict subjects with depression (Supplementary Figure 1C, red). Finally, we assessed the distribution of electrodes that were assigned to each module across the main anatomical regions defined by Cammoun et al. (2012) (Supplementary Figure 1D).

### Assigning Names to Modules

We assigned a name to each module by examining the location of each module’s most influential electrodes. We utilized the participation coefficient (PaC), which is a degree-based measure of network connectivity that describes a node’s functional interaction within and across network modules (Guimera and Amaral, 2005; Rubinov and Sporns, 2010; Bertolero et al., 2015). This metric is typically utilized to identify influential hubs across a large-scale network. We utilized it in our study to identify the location of hubs that were most important for driving connectivity in each module identified through community detection. Groups of electrodes with low PaC values indicate hubs with high intramodular connectivity, also known as provincial hubs (van den Heuvel and Sporns, 2013). Similarly, connector hubs are those with high PaCs and drive intermodular connectivity. The PaC describes the weight of edges from node i to all other nodes in the same module relative to the weight of edges from that node to all nodes in the network according to


(3) $$y_{i}=1-\sum_{c\in C}\left(\frac{k_{i}\left(c\right)}{k_{i}}\right)$$

where y_*i*_ is node *i*′s participation coefficient, C is the set of all modules, *k*_*i*_(*c*) is the sum of all correlations between node *i* and other members of module C and *k*_*i*_is the sum of all correlations between node *i* and members of all modules. We calculated the PaC for each electrode across our model, and then selected those with high and low participation values (top/bottom 10%). We then grouped these selected nodes by Lausanne atlas region, eliminating or combining a minority of regions due to having too few electrodes for analysis. We addressed the non-uniform distribution of electrodes across the model by then assigning each Lausanne region a score according to the following hub weight:


(4) $$R_{i}=\frac{N_{i}M_{j}}{T_{j}}$$

where *N*_*i*_is the number of selected electrodes (top/bottom 10%) in Lausanne region *i*, *M_j_* is the number of selected electrodes in Lausanne region *i* of module *j*, and *T*_*j*_ is the number of total electrodes across modules in Lausanne region *i*. Hubs were those Lausanne regions with the highest hub weight. Hub location was identified by averaging the MNI coordinates of electrodes within each hub. The full list of Lausanne regions and hub weights is shown in Supplementary Figure 2 and Supplementary Tables 2, 3. The purpose of the identified hubs in the present report was primarily descriptive and helped relate the computational model to known brain regions and structure; all subsequent analyses utilized the population set of electrodes across the full model.

### Model Utilization: Activity Analysis

We next used the whole-brain correlational model to relate the architecture and intrinsic neural activity of functional networks to depression status. We hypothesized that by leveraging the high temporal resolution of iEEG, as well as the direct access to subcortical structures, we could overcome limitations of scalp recordings (Widge et al., 2019). We used a machine learning algorithm validated with leave-one-out cross validation to identify distributed neural circuit features that discriminated depression. We first averaged local field potentials across the electrodes within each module and then decomposed the signals into common spectral bands to identify 36 features (6 frequency bands × 6 modules) where each feature contained information about a spectral power band across one functional module. These features, referred to as spectral-spatial features, served as our starting feature space for entry into our classification pipeline. Transformation with principal component analysis (PCA) (Hotelling, 1933) followed by methods for feature selection and subsequent discrimination have been used on previous iEEG classification problems (Kirkby et al., 2018; Sani et al., 2018). We followed a similar pipeline. PCA enabled us to identify a low-dimensional feature representation of spectrally band-limited neural activity across electrodes that potentially span different modules. It is important to note that while PCA and network module detection reduce the complexity and inherent collinearity in the dataset (Manning et al., 2011, 2012; Kirkby et al., 2018; Sani et al., 2018; Scangos et al., 2019b), they also reflect two non-mutually exclusive properties of brain connectivity (modules) and brain activity (principal components). Specifically, modules demarcate groups of brain regions with correlated *broadband* brain activity, irrespective of the amplitude of the activity, and principal components represent additional state-dependent neural activity that is *band-specific*, such as rhythms and oscillations (Betzel et al., 2019), and may arise from functionally important integrative connections that span between modules (Bertolero et al., 2015; Betzel et al., 2018). This line of thinking closely resembles previously reported accounts of neural co-activation dynamics (akin to principal components) spanning multiple cognitive networks (akin to network modules) that explain inter-individual differences in task performance and cognitive traits. After identifying a principal component representation of cross-module spectral-spatial network features, we utilized logistic regression (with L1 regularization) to classify subjects with depression and identify features with the greatest discriminatory power. PCA and logistic classification were performed within the cross-validation loop where a model is trained on all subjects but one, and then tested on the held-out subject with each subject held-out in turn. We report mean accuracy (balanced to group-size) across the cross-validation iterations. Models without PCA were also performed for comparison (L1, L2, elastic net, random forest). To further asses our model validity, we repeated our classification pipeline on a null model obtained from randomly permuting the target class labels 1,000 times and used a permutation test to assess significance between the true and null model accuracy distributions. In order to control for possible differences in epileptiform activity residual to data-cleaning across the modules we calculated mean line-length, a commonly utilized measure for the detection of epileptiform activity (Guo et al., 2010), of the electrodes within each module and used a logistic regression model to determine if line-length across the six modules was a significant predictor of depression status.

### Hierarchical Clustering to Identify Depression Networks

We reasoned that we could utilize the group-level network to identify common features that defined depression at the individual level. To do so, we mapped the principal component values (feature loadings ≥ 0.2) back to the original feature space weighted by the logistic regression coefficients. Specifically, we computed the dot product between the loading weights for each spectral-spatial feature and the coefficient weighting from the classifier. Performing this operation provided the log-odds impact of each original feature and enabled us to show the direction of change of each power band and module in relation to depression diagnosis. We then tested the distribution of feature impact on depression classification probability across depressed participants by grouping similar log-odds impact covariates (thresholded at 0.15) utilizing an agglomerative hierarchical clustering algorithm (Ravasz et al., 2002; Rihel et al., 2010; Drysdale et al., 2017; Grisanzio et al., 2018). A log-odds threshold of 0.15 was selected because it retained classification results for 98% of subjects while isolating the most contributory spectral-spatial features (see Supplementary Figure 3A for non-thresholded model for comparison). The clustering yielded both patient and feature groupings that defined neurophysiological network expression patterns (NEPs) of depression. We quantified the impact of these NEPs on each participant’s probability of being classified as depressed by performing a sensitivity analysis where we withheld each NEP and then attributed the probability decrement from the total classification probability to the withheld activity pattern. We also ran this analysis on the boundary patients who had mild symptoms of depression but did not reach threshold (PHQ-9 < 10) for depression (Supplementary Figure 3B).

### Model Utilization: Connectivity Analysis

In addition to alterations in the spectral content of network activity in depression, previous studies have observed distinct deficiencies in connectivity across depression networks (Sun et al., 2011; Zhang et al., 2011; Lord et al., 2012; Korgaonkar et al., 2014; Chen et al., 2017). A fundamental interest in neuroscience is the relationship between the brain’s neural activity and its underlying functional and structural connectivity, which remains unknown. The graph of our whole-brain iEEG model defines correlational relationships between electrodes across our total population. We thus examined these correlational relationships across control and depressed groups independently in order to measure the relative differences of functional network organization between the two groups. First, inter- and intramodular connectivity strengths were assessed by looking at the correlations between all electrodes within the same module (intramodular) and the correlations between electrodes across all pairs of modules (intermodular). Next, to assess whether the effect of connectivity differences between groups is a network-wide characteristic of the depressed brain or whether the effect is localizable to specific modules, we used a Cohen’s d effect size metric and compared the distribution of correlation strengths across depressed and control groups for each possible module pair. To assess significance across these connections we generated a null distribution of Cohen’s *d* values for each module pair and retained the true Cohen’s *d* values that survived multiple comparisons testing (*p* < 0.001).

## Results

### Derivation of Functional Modules

**FIGURE 3:**
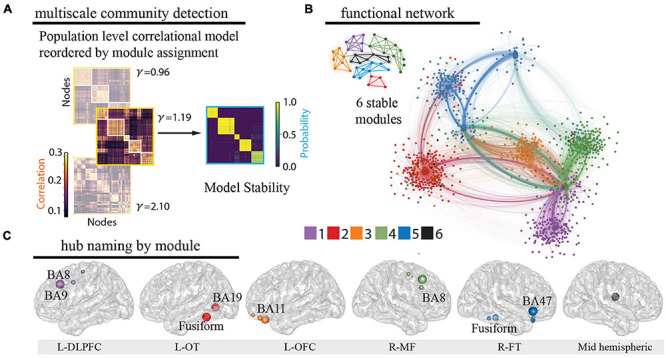
Identification of functional modules. **(A)** Multiscale community detection was applied to the whole-brain model to group electrodes (nodes) into non-overlapping modules (communities) by their correlational relationships (Newman, 2006; Blondel et al., 2008). First, the population-level correlational model was reordered by the module assignment according to the modularity cost function. Network modules were identified at different levels of granularity by varying the tuning parameter (Garcia et al., 2018; He et al., 2018). Increasing partitions the brain into increasing numbers of modules with a limit equal to the number of electrodes, as shown here for 3 values of (left). Next, the stability of this clustering at each value of was assessed by calculating module allegiance, which describes the probability that any two electrodes occupy the same module on repeated module detection (Bassett et al., 2015) (right). A value of 1.19 was selected by comparing the similarity of partitions generated by values of with those of a commonly used brain atlas (Cammoun et al., 2012), resulting in 6 modules. Of note, one of the modules is small and difficult to resolve in the figure. **(B)** The graph of the large-scale network with module membership delineated by the color of the nodes and edges for selected of 1.19 is shown along with a schematic representation of the 6 modules. **(C)** Hubs for each module were identified by selecting electrodes with the lowest 10% participation coefficients. Values were then averaged for each Lausanne brain region per module and weighted by the distribution of electrodes across Lausanne regions in all modules. Hub weight is indicated by the size of hub, and module assignment is indicated by hub color. Module 5 contained insufficient number of electrodes for hub identification (0.3% of total sample) and coefficients across all electrodes were utilized to name this module. L-DLPFC, left dorsolateral prefrontal cortex; L-OT, left occipitotemporal cortex; L-OFC, left orbitofrontal cortex; R-MF, right medial frontal cortex; R-FT, right frontotemporal cortex.

Using leave-one-patient out validation of the correlational model, we found that the distribution of correlations (mean *r* = 0.38) was similar to the prior reconstruction accuracies (Owen et al., 2020) and centered well above shuffled correlational models (mean *r* = 0.00) suggesting the algorithm estimates activity patterns substantially better than chance. The distribution of patient level fisher transformed correlation coefficients was significantly different than 0 (*t* = 13.94, *p* = 1.04e^–25^, Figure 2F). We observed that our whole-brain iEEG model was optimally parcellated into 6 stable modules (Jaccard index, *p* < 0.05, permutation test) and that these modules were spatially distributed and spanned multiple anatomical structures (Figure 3A). A graph of the network and its subdivision into modules is shown in Figure 3B, where module membership is indicated by the color of nodes (iEEG electrodes) and edges (inter-electrode correlation from whole-brain model). These modules included the left dorsolateral prefrontal cortical (L-DLPFC), left occipitotemporal (L-OT), left orbitofrontal cortical (L-OFC), right frontotemporal (R-FT), right medial frontal (R-MF), and mid-hemispheric modules. Figure 3C shows hub locations by their mean Montreal Neurological Institute (MNI) (Cammoun et al., 2012) coordinates and associated Brodmann Areas.

### Relationship of Functional Network Identification to Depression Status

**FIGURE 4:**
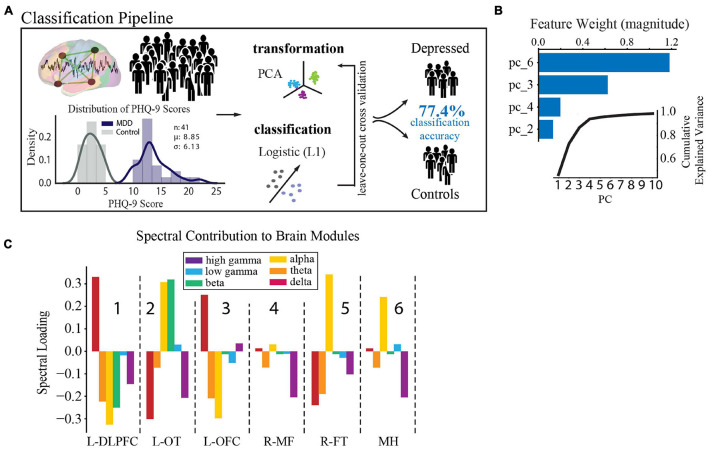
Spectral-spatial features that discriminate depression at group level. **(A)** Activity analysis pipeline showing steps including power feature extraction, dimensionality reduction, transformation, and classification. The distribution of PHQ-9 scores across the depression (*n* = 18, purple) and control groups (*n* = 23, gray) is shown bottom left (mean PHQ-9 score 8.85, standard deviation 6.13). Power was extracted from the reconstructed time-series using the Morlet transformation in 30 s intervals across 6 frequency bands (delta = 1–4 Hz, theta = 5–8 Hz, alpha = 9–12 Hz, beta = 13–30 Hz, low gamma (gammaL) = 31–70 Hz, high gamma (gammaH) = 71–150 Hz). This process yielded 25,464 spectral power features from our model (6 frequency bands × 4,244 electrodes × 2 h). Z-scored relative power was calculated and averaged within each band across each of the 6 network modules. Power was then further averaged across time to yield 36 spectral-spatial features per participant. Principal component analysis was then used to transform the full spectral-spatial feature set, followed by logistic classification yielding 4 features that identified depression with 80.0% accuracy on the training set and 77.4% on the test set. **(B)** The component weights of the four features with cumulative explained variance across the first 10 principal components shown in the inset. **(C)** Spectral distribution of the 4 components was obtained by calculating the dot product between the loading weights (>0.2) for each spectral-spatial feature in the four principal components and the coefficient weighting from the classifier. Bars show the direction of change of each power band and module in relation to depression diagnosis and relate changes in spectral power associated with depression across spatially distributed brain networks. These spectral-spatial features represent the circuit activity that distinguishes depression in our population. DLPFC, dorsolateral prefrontal cortex; OT, occipitotemporal; OFC, orbitofrontal cortex; MF, medial frontal; FT, frontotemporal; MH, mid-hemispheric.

In accordance with literature-derived rates of depression in this population (Hermann et al., 2000; Gilliam et al., 2003; Swinkels et al., 2005; Hermann and Jones, 2006; Fuller-Thomson and Brennenstuhl, 2009; Rai et al., 2012), 43% of our population had self-reported depression (defined by PHQ-9 ≥ 10, *n* = 23), and 33% had mild or no symptoms of depression, which defined our control group (PHQ-9 ≤ 5, *n* = 18). The two groups did not vary in age, sex, type of epilepsy, antidepressant usage, or anti-epileptic drug class (*t*-test, X^2^, *p* > 0.4, Supplementary Table 1). In order to determine the spectral-spatial neural activity features that discriminated the depressed from the control group, we used a standard leave-one-out cross validated machine learning pipeline (PCA followed by logistic regression, Figure 4A) (Arbabshirani et al., 2017). We found that a combination of four principal components had the strongest predictive ability to detect depressed from non-depressed subjects. Their loading weights represent their contribution toward likelihood of depression (Figure 4B). Utilizing the four most discriminative components alone, we achieved a mean classification accuracy of 77.4% (*p* = 0.002). The same classification pipeline applied to a null model obtained from randomly permuting the target class labels 1,000 times and retraining the classifier with each permutation led to an accuracy of 50.0%. Alternate classification models without PCA also performed better than chance (L1 0.68; L2 0.77; Elastic Net 0.75; Random Forest 0.60). Furthermore, a logistic regression model showed that epileptiform activity residual to data-cleaning across the modules was not a significant predictor of depression status (*R*^2^ = 0.15, *p* = 0.13). Together, these data suggest that a parsimonious model with four principal components, which capture major sources of variance in spectral-spatial features, can detect subjects with depression from the control group significantly better than chance.

As our primary goal was to uncover the underlying biology of depression, we next turned to an examination of the individual spectral-spatial features contained within the four components. These features comprise the circuit activity that distinguishes depression in our population (for full component loadings see Supplementary Table 4). To better interpret the biological meaning of this distributed network activity in terms of recognized brain regions and our similarly scaled network modules, we spatially projected the four components back onto the brain (Figure 4C). On visual inspection two gross patterns of spectral activity across the modules emerged. The first was high alpha power across the L-OT, R-FT, and mid-hemispheric modules (attention and default mode regions, modules 2,5, and 6 in Figure 4C). The second was high delta and low alpha and theta power in the L-DLPFC and OFC modules (executive and limbic regions, modules 1 and 3 in Figure 4C). These results suggested that low- and mid- frequency activity across broad networks characterize depression at the group level and motivated the subsequent statistical analysis to define the two patterns quantitatively.

### Distinct Network Expression Patterns Define Depression

**FIGURE 5:**
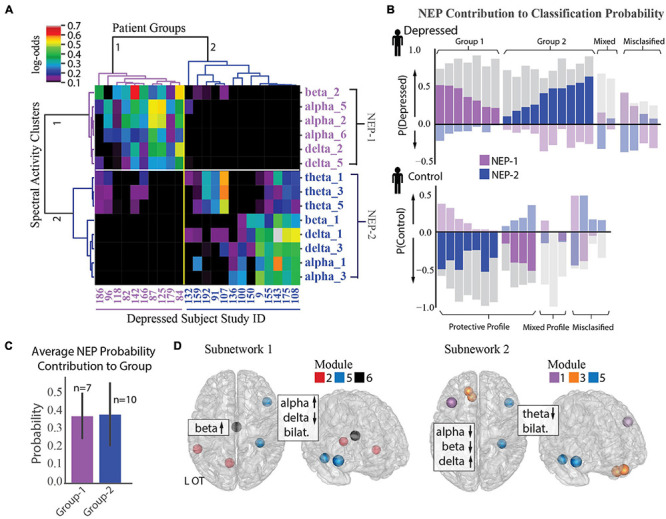
Identification of two depression subnetworks. **(A)** Hierarchical clustering on log-odds of spectral-spatial features at the individual patient level showing 2 patient groups (horizontal groupings) and 2 network expression patterns (NEPs) (vertical groupings). Columns represent individual patients with patient study number shown at bottom, and rows represent spectral power across one frequency band and module (ex. alpha_1 = alpha power across module 1). Magnitude of log-odds represented by color of corresponding boxes (color-bar legend top right). Spectral-spatial features associated with NEP-1 represented in purple text and those associated with NEP-2 represented in blue text. **(B)** NEP probability contribution for the depressed group (top plot) and control group (bottom plot) derived from a sensitivity analysis where the probability of depression for each individual was calculated in total and with a perturbation where each NEP was held out. The probability difference was attributed to the presence of the NEP. This probability contribution is represented by the colored bars overlaid over each patient’s total probability of being depressed as derived from the machine learning classification model (gray bars, probability > 0.5 leads to classification of depression). The perturbations do not sum to produce the total classification probability; rather each quantifies the relative importance of that NEP toward depression. Bars in the positive direction indicates a positive contribution toward depression, and those in the negative direction indicate a protective contribution toward depression. Subjects where one of the two NEPs did not drive classification probability are shown in muted colors (mixed profile). Subjects classified incorrectly shown on far right of each plot (misclassified). **(C)** Mean probability contribution of each NEP to two patient groups is shown. NEP-1 (purple bars) contributed most strongly to the probability of depression in the first group (mean = 38% probability contribution, SD = 0.13) and NEP-2 (blue bars) contributed most strongly to a second group (mean = 39% probability contribution, SE = 0.18). Number of participants who exhibit each NEP shown above each bar. Error bar = standard deviation. **(D)** Direction of activity and spatial distribution of activity changes within NEP shown on glass-brain in several orientations. Hubs for each module within the NEP are designated by hub color.

To further examine the observed inter-individual heterogeneity in expression of the group-level depression network features, we tested the distribution of feature impact on depression classification probability across participants using an agglomerative hierarchical clustering algorithm (Ravasz et al., 2002; Rihel et al., 2010; Drysdale et al., 2017; Grisanzio et al., 2018). We found two distinct subnetwork activity patterns (network expression patterns (NEPs)) that strongly impacted depression and subdivided our depressed population into two groups (Figure 5A). The first subnetwork (NEP1) was marked by increased beta power in the L-OT module, and increased alpha and decreased delta power over the L-OT and R-FT modules. The second subnetwork (NEP2) was marked by decreased theta in the L-DLPFC, L-OFC, and R-FT modules, and decreased alpha, beta power together with increased delta power within the L-DLPFC and L-OFC modules. The presence of two subnetworks importantly demonstrated that different core features were relevant in different subjects.

We next used a sensitivity analysis to quantify the impact of each NEP on each participant’s probability of being classified as depressed. Figure 5B shows the probability contribution of each NEP for each subject in the depressed group (top plot) and control group (bottom plot). While we anticipated that each individual would exhibit several NEPs with differing contributions to their depression classification, an alternate pattern emerged from the data. We found that increased activity in either NEP was correlated with depression, but that each patient exhibited activity in only one of the two NEPs. Thus, depressed participants fell into two groupings based on NEP activity. Classification for the first group (37% depressed subjects) was largely driven by NEP1 (*n* = 7, mean probability contribution = 0.38, SD = 0.13) alongside usually modest opposing contributions form NEP2, while classification for the second group (53% depressed subjects) was largely driven by NEP2 (*n* = 10, mean probability contribution = 0.39, SD = 0.18, Figure 5C), alongside more modest opposing contributions from NEP1. Classification of the remaining 11% of participants was either driven by mixed effects of both NEPs or there was little contribution from either NEP and may be evidence of additional subnetworks that were not resolved in our dataset. Two distinct groups also emerged from the control participants with NEP activity contributing here as well, but with distinct contribution profiles compared to the depressed participants. Classification for the first group (21% control patients) was driven either by mixed effects of both NEPs or little contribution of either NEP, as we anticipated. Classification for the second group, was driven by one of the two NEPs with a more modest contribution of the opposing NEP (79% of control group). We might speculate that relative NEP activity could represent either risky or conversely, protective activity profiles, and that NEP activity could be modulated in either direction to treat depression. The anatomical distribution of the two depression subnetworks and the associated changes in spectral activity are shown in Figure 5D and Supplementary Figure 3C.

### Network Organization Is Disrupted Across Depression Subnetworks

**FIGURE 6:**
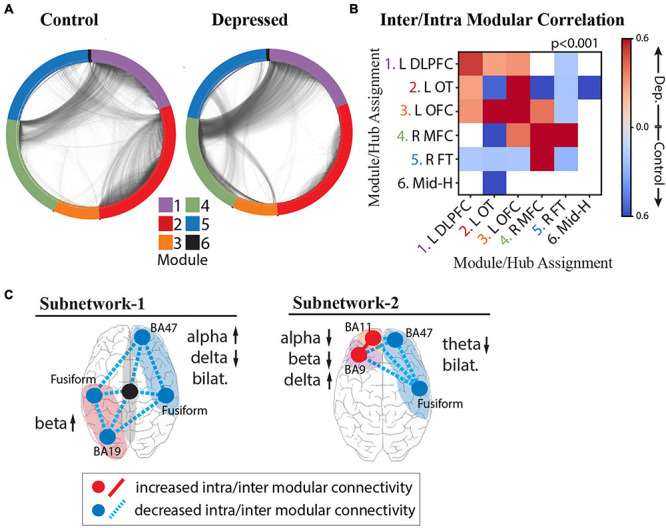
Intra- and Inter-modular connectivity signatures of depression and control groups. **(A)** Connectivity structure derived using whole-brain iEEG model recalculated for the control group (left) and depressed group (right) with module membership delineated by the color of the nodes (electrodes), and edges (connections between electrodes) delineated by the black interconnecting lines. **(B)** Heatmap of significant Cohen’s *d* values calculated from the distribution of correlation strengths between depressed and control groups for each possible module pair and compared to Cohen’s *d* values for a null distribution derived from permuted nodal module assignment. Those that survived multiple comparison testing (*p* < 0.001) were retained (red: increased connectivity for depressed group; blue: increased connectivity for control group; white: not significant). **(C)** Schematic of NEP-1 (left) and NEP-2 (right) showing both connectivity and spectral power underlying each pattern. Increased connectivity strength shown in red, and decreased connectivity shown in blue (hub = intramodular, line = intermodular connectivity). Color of shaded area refers to module number as shown in color legend in **(A)**.

We expected that alterations in functional network topology would also be present in our depressed population and that we could delineate new relationships between activity and functional connectivity with our high-resolution dataset to more comprehensively characterize depression subnetworks. We performed a connectivity analysis using correlation of local field potential activity across modules as an estimate of functional connectivity between electrodes. Figure 6A shows the two-dimensional representation of the functional network structure for control (left) and depressed (right) groups. In comparison to the control group, we qualitatively observed an overall reduction in the segregation between modules in the depression network.

To quantify these differences and test whether the effect of connectivity differences between groups is a network-wide characteristic of the depressed brain or whether the effect is localizable to specific modules, we calculated the inter- and intra-modular connectivity strength. Figure 6B shows the heatmap of significant Cohen’s *d* values, where a greater effect of connectivity for the depressed group is indicated in red, and lower effect of connectivity for the depressed group is indicated in blue. The results demonstrate strong evidence that, indeed, there are module-specific differences in the effect of connectivity between depressed and non-depressed individuals suggesting that modules may express hyperconnectivity or hypoconnectivity in depression depending on their anatomical localization in the brain. In the depressed group, there was overall greater frontal connectivity and weaker cross-hemispheric connectivity. Specifically, we observed greater intra-modular connectivity within L-DLPFC, L-OFC, and R-MFC modules, weaker intra-modular connectivity within L-OT and R-FT modules, and greater inter-modular connectivity between L-DLPFC, L-OFC, and L-OT modules. Hubs in the insula, amygdala, temporal pole and fusiform gyrus drove the cross-module connectivity (top 10% participation coefficient, see section “Materials and Methods”). We also observed a decrease in cross hemispheric connectivity in the depressed group compared to the control group (L-DLPFC/L-OFC to R-FT modules, and L-OT to R-FT/R-MFC modules), with hubs in the insula, temporal-parietal region and amygdala responsible for this decreased connectivity. The L-OFC module showed greater connectivity with the R-MFC module, and R-MFC module exhibited stronger connectivity with the R-FT module.

On the basis of the above analyses we were able to parse specific connectivity components that characterize the two depression subnetworks (Figure 6C), unifying both activity and connectivity analyses across cortical and deep structures with a level of specificity that has not previously been possible. In the first subnetwork characterized by NEP1 we observed increased beta power in the L-OT module, and right-left asymmetry in the alpha and delta bands over right frontal/L-OT modules with weaker intra- and inter-modular connectivity throughout. In the second subnetwork characterized by NEP2 we observed a hyperactive left frontal cortex that was more highly connected within itself but more weakly connected to R-FT module. Lower theta bilaterally was observed in this subnetwork.

## Discussion

In this report, we present a large study of direct neural recordings aimed at identifying depression networks, made possible by multi-day iEEG recordings paired with a depression measure. The opportunity to directly record semi-chronically from cortical and subcortical structures in this manner enabled us to estimate whole-brain neural activity and incorporate both activity and connectivity analyses to resolve new subnetworks underlying depression. We found that depression is associated with a complex distributed pattern of network activity and two distinct depression subnetworks were expressed in 89% of depressed patients. These included a poorly connected occipitotemporal network characterized by heightened beta activity, and a hyperconnected frontal cortical subnetwork characterized by low alpha and theta power.

Our ability to delineate the functional organization and spectral activity patterns of depression networks with high spatiotemporal resolution relied on the application of a network neuroscience framework to the output of the SuperEEG model. Recently, Betzel and colleagues successfully applied a similar correlational network model to multi-subject iEEG recordings, followed by community detection, and found network organization to be representative of that obtained from DTI and fMRI (Betzel et al., 2019). We further extended these findings, by applying the iEEG model to the study of disease status for the first time. The two depression subnetworks we identified are supported by previous fMRI and EEG studies of depression that have found individual components of the subnetworks in different studies including limbic alpha power that correlates with depression severity (Neumann et al., 2014), disruptions in frontal theta, temporal beta (Newson and Thiagarajan, 2018), and alpha asymmetry (Henriques and Davidson, 1990, 1991; Tomarken et al., 1992; Wheeler et al., 1993; Gotlib et al., 1998). Decreased connectivity in the occipital, temporal, and right medial frontal regions (Veer et al., 2010) and higher frontal connectivity has also been observed (Nofzinger et al., 2005; Greicius et al., 2007; Frodl et al., 2010; Sheline et al., 2010; Alexopoulos et al., 2012; Cheng et al., 2016). Our findings of two dichotomously expressed subnetworks may provide a partial explanation for the inconsistent findings across prior EEG studies that have predominantly focused on single frequency band or brain regions and have lacked rigorous cross-validation as noted by a recent meta-analysis (Widge et al., 2019).

Prior analyses of neuropsychiatric-related iEEG features have been made using components of the patient dataset used in this study (Kirkby et al., 2018; Sani et al., 2018; Scangos et al., 2019a). These efforts (Kirkby et al., 2018; Sani et al., 2018) have focused on studying a broad emotion state rather than depression and took region-based approaches using low subject numbers due to the problem of heterogenous electrode coverage across individuals. The computational approach developed here was motivated by limitations of this prior work, enabling us to incorporate parallel information from all of our subjects despite differing electrode coverage, perform group level analyses of depression, and uncover distributed circuit activity. While our aim was to capture network dysfunction associated with depression, the two distinct ways in which activity within the NEP networks combinatorically relates to disease classification also suggest the possibility of their reflecting depression biotypes. Deeper exploration of these putative biotypes awaits further study.

Functional connectivity informs longer time-scale organization of neural populations whereas functional activity informs moment-to-moment behavior of neural populations. Our finding that some brain regions show distinct changes in both activity and connectivity, while other regions, such as the right medial frontal region (module 4), demonstrate connectivity differences alone suggests that depression is both a state-invariant connectivity disorder and a state-dependent activity disorder. This relationship might explain why traditional antidepressant medications can take 6–8 wks to start working, yet ketamine can improve symptoms on the same day of administration (McGirr et al., 2015). It is possible that the presence of aberrant activity over long periods of time could shape network connectivity via plasticity or that changed connectivity patterns can impact the timing and flow of normal neural activity. Future work using high temporal resolution iEEG could inform how symptom-states and depression traits are integrated at the level of distributed neural circuits.

We acknowledge some weaknesses in the results presented. Depression in epilepsy is thought to arise from similar origins to primary depression [ex. stress (Wulsin et al., 2016), inflammation (Vezzani et al., 2011), circuit dysfunction (Gleichgerrcht et al., 2015)], and is responsive to antidepressants (Kanner, 2003) suggesting it can provide valuable insight into depression more broadly. It remains unknown whether the depression networks we identified are related to the presence of epilepsy. Our categorical approach using the PHQ-9 to identify depressed patients was straightforward to apply in the context of complex data and has direct clinical relevance. However, it also selects inherently imperfect diagnostic boundaries and limited our capacity to examine variation in depression among subjects. Furthermore, as this was a cross-sectional investigation, some patients in the control group had a history of depression treated with ongoing antidepressant use but were not depressed per the PHQ-9 at the time of the study. Future analyses could explore how neural signatures vary with symptom severity in addition to alternative dimensional approaches which have the potential benefit of mapping neural features onto symptom profiles (Drysdale et al., 2017; Grisanzio et al., 2018). Furthermore, assumptions about the number of communities are a limitation of the community detection method (Betzel et al., 2019). Future studies could explore changes in network structure across depressed and non-depressed individuals at different levels of resolution.

While our whole-brain iEEG model was extensive in coverage, we did not have electrodes placed in all brain regions, including some regions implicated in depression (Mayberg et al., 1997; Malone et al., 2009; Hamani et al., 2011; Marchand et al., 2012; Riva-Posse et al., 2018) and the density of electrode sampling varied across brain regions leading to uncertainty in the accuracy of estimation in sparsely sampled areas (Owen et al., 2020). We dealt with this constraint by discounting the effect of each individual node degree before running community detection and comparing network measures to a null model that accounted for overall node density. Furthermore, our prior work has shown no reliable correlation between reconstruction accuracy and density (Owen et al., 2020). SuperEEG relies on accurate reconstruction of held-out activity patterns. While accuracy of this algorithm is significantly above chance and similar to the test-retest reliability of fMRI in redetecting estimated activity (Bennett and Miller, 2010), improved reconstruction is an important area for future work. The SuperEEG approach reconstructs just a portion of the verum iEEG signal – the remaining unexplained portion may stem from subject-specific variation in connectivity (Mueller et al., 2013; Finn et al., 2015), state-dependent variability in connectivity (Hutchison et al., 2013a, b) within subjects, or statistical noise. It follows that of this faithfully reconstructed portion of the iEEG signal, we found that higher-order principal components of spectral-spatial iEEG activity were most important for identifying patients with depression. Taken together, we speculate that depression may in fact have a low-dimensional network representation that is widely pervasive in the iEEG signal but represents just a small portion of iEEG signal dynamics. Importantly, we found that alternate machine-learning pipelines converged on these same low-dimensional features. Thus, there is high likelihood that the neural features we have found reflect circuit physiology that is stereotyped to depression.

With advancements in data processing capabilities and accessibility we may be able to reduce assumptions and the estimation burden, extend coverage to more brain regions, and utilize larger samples. Indeed, work to integrate our findings with network features from high spatial resolution MRI is already underway by our group. Finally, while ideally we would have independent test and training datasets for the machine learning used for classification, we utilized leave-one-out cross validation due to our sample size.

Through the current study, we identified two novel subnetworks of depression. The results have important implications for disease subtyping, diagnosis, treatment planning, and monitoring of depression status. These subnetworks could form the basis for interventions at many different potential control points along each subnetwork and suggest that interventions that change both connectivity and spectral power could be promising. For example, they provide a mechanistic rationale for practitioner’s choice between right and left DLPFC vs. OFC targets for repetitive transcranial magnetic stimulation (Drysdale et al., 2017; Feffer et al., 2018). Evidence of high activity in one network pattern, countered by an anti-weighting of the other pattern further suggests the existence of protective or high-risk profiles and the possibility of preventative treatments. A library of new treatment targets and frequency-specific treatment parameters (Chanes et al., 2013; Cocchi and Zalesky, 2018) could enable a new wave of interventional therapies that personalize treatment based on neurophysiological signals.

## Data Availability Statement

The raw data supporting the conclusions of this article will be made available by the authors, without undue reservation. The electrophysiological whole-brain atlas (correlational model) has been uploaded to zenodo (https://zenodo.org/record/5540172#.YVUn9WZKjzc).

## Ethics Statement

The studies involving human participants were reviewed and approved by the Institutional Review Board at University of California, San Francisco. The patients/participants provided their written informed consent to participate in this study.

## Author Contributions

KS, AKh, EC, and AKr conceived the study. KS, AKh, and PD analyzed and interpreted the data. JA and EA contributed to data collection. JM and LO contributed to data analysis methods. KS wrote the manuscript with significant input from all authors. All authors reviewed and approved the manuscript.

## Conflict of Interest

AKr consults for Eisai, Evecxia, Ferring, Galderma, Harmony Biosciences, Idorsia, Jazz, Janssen, Merck, Neurocrine, Pernix, Sage, and Takeda. EC has patents related to brain stimulation for neuropsychiatric conditions, brain mapping, and speech neuroprosthesis and also given talks related to epilepsy treatment for Neuropace and Cyberonics/Livanova. The remaining authors declare that the research was conducted in the absence of any commercial or financial relationships that could be construed as a potential conflict of interest.

## Publisher’s Note

All claims expressed in this article are solely those of the authors and do not necessarily represent those of their affiliated organizations, or those of the publisher, the editors and the reviewers. Any product that may be evaluated in this article, or claim that may be made by its manufacturer, is not guaranteed or endorsed by the publisher.
