# Supplementary figures and images for: Distributed Subnetworks of Depression Defined by Direct Intracranial Neurophysiology
Source: Front Hum Neurosci. 2021 Oct 21;15:746499. doi: 10.3389/fnhum.2021.746499 (PMC8566975; doi:10.3389/fnhum.2021.746499)

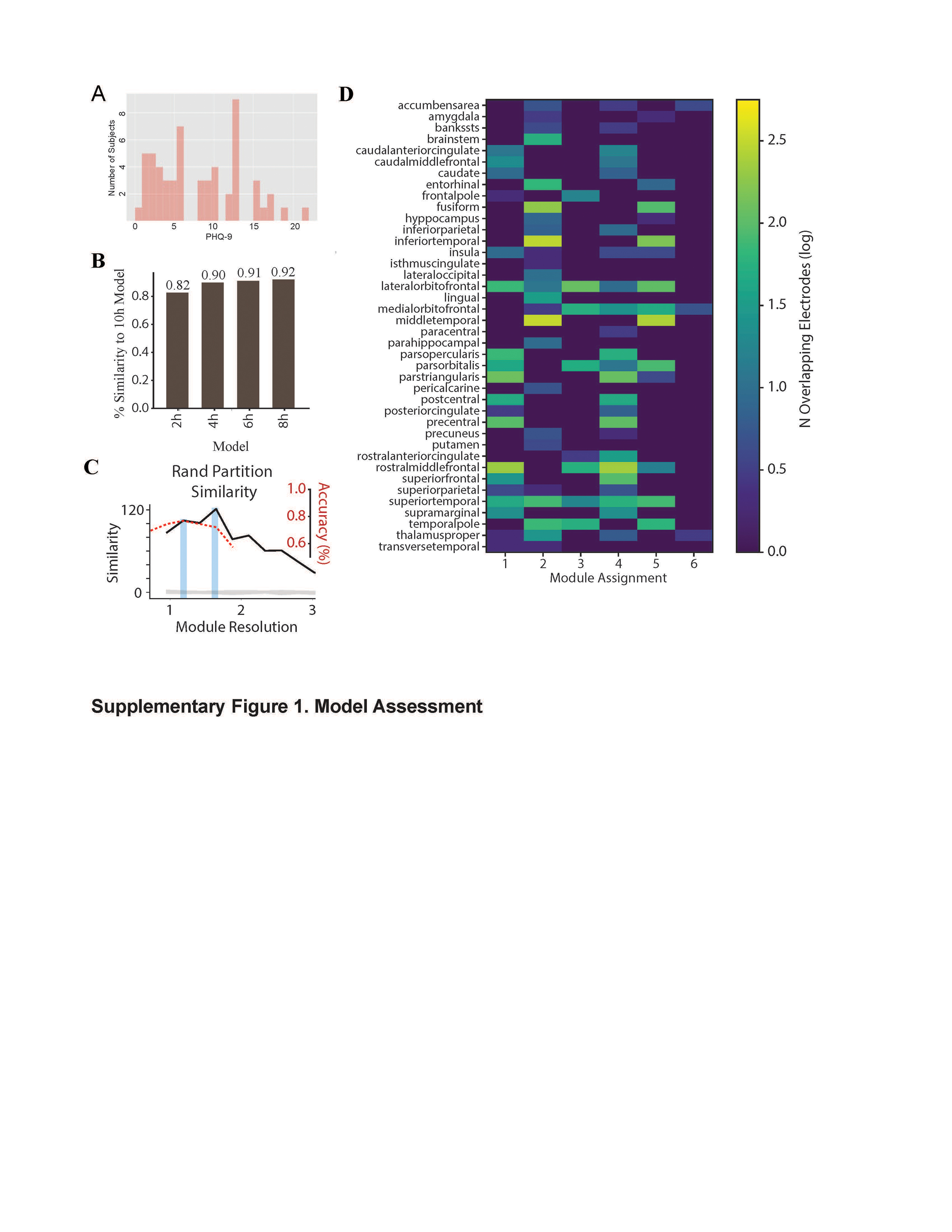

Supplement: Supplementary file 2 [file Image_1.jpeg]

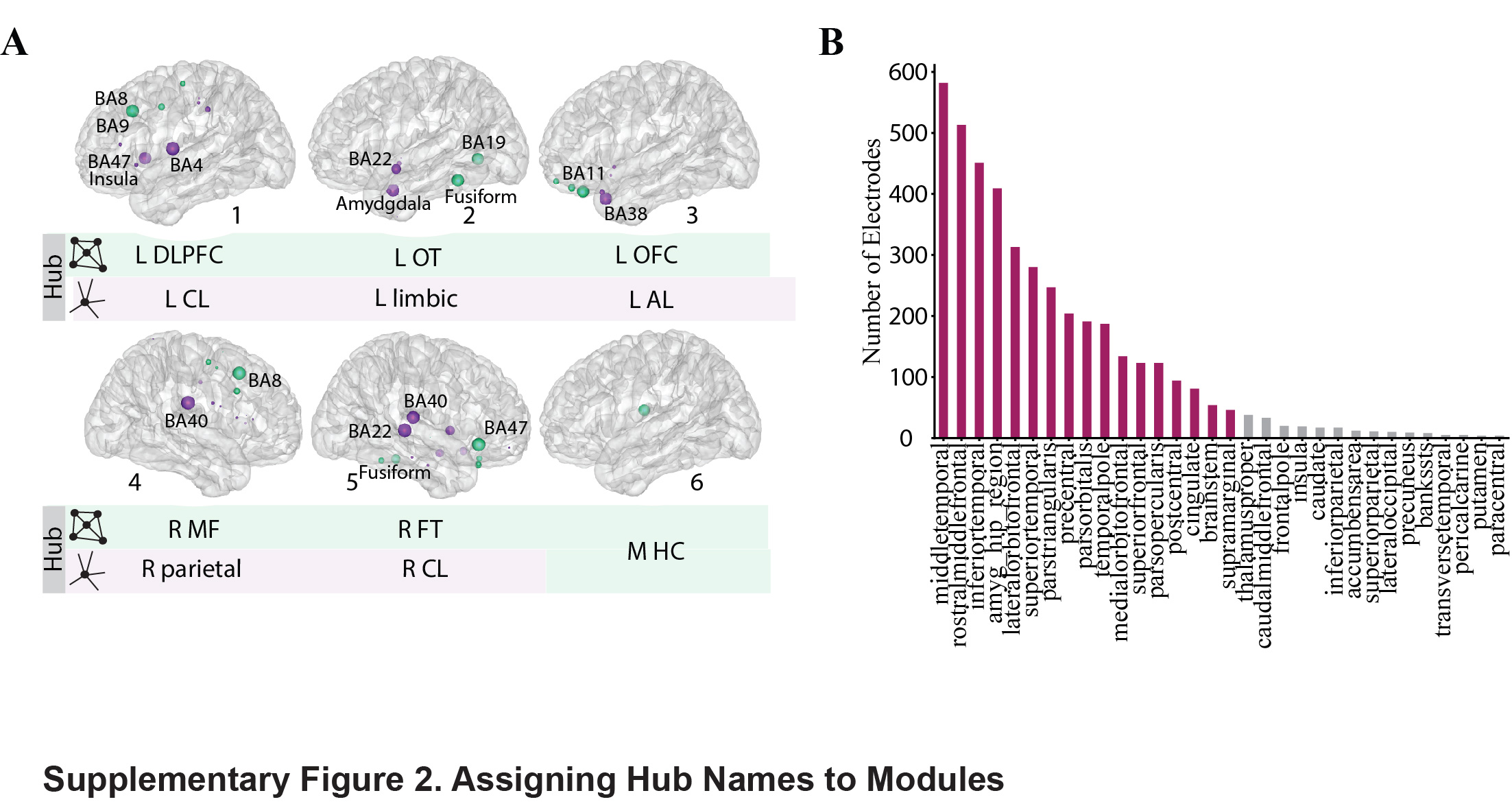

Supplement: Supplementary file 3 [file Image_2.jpeg]

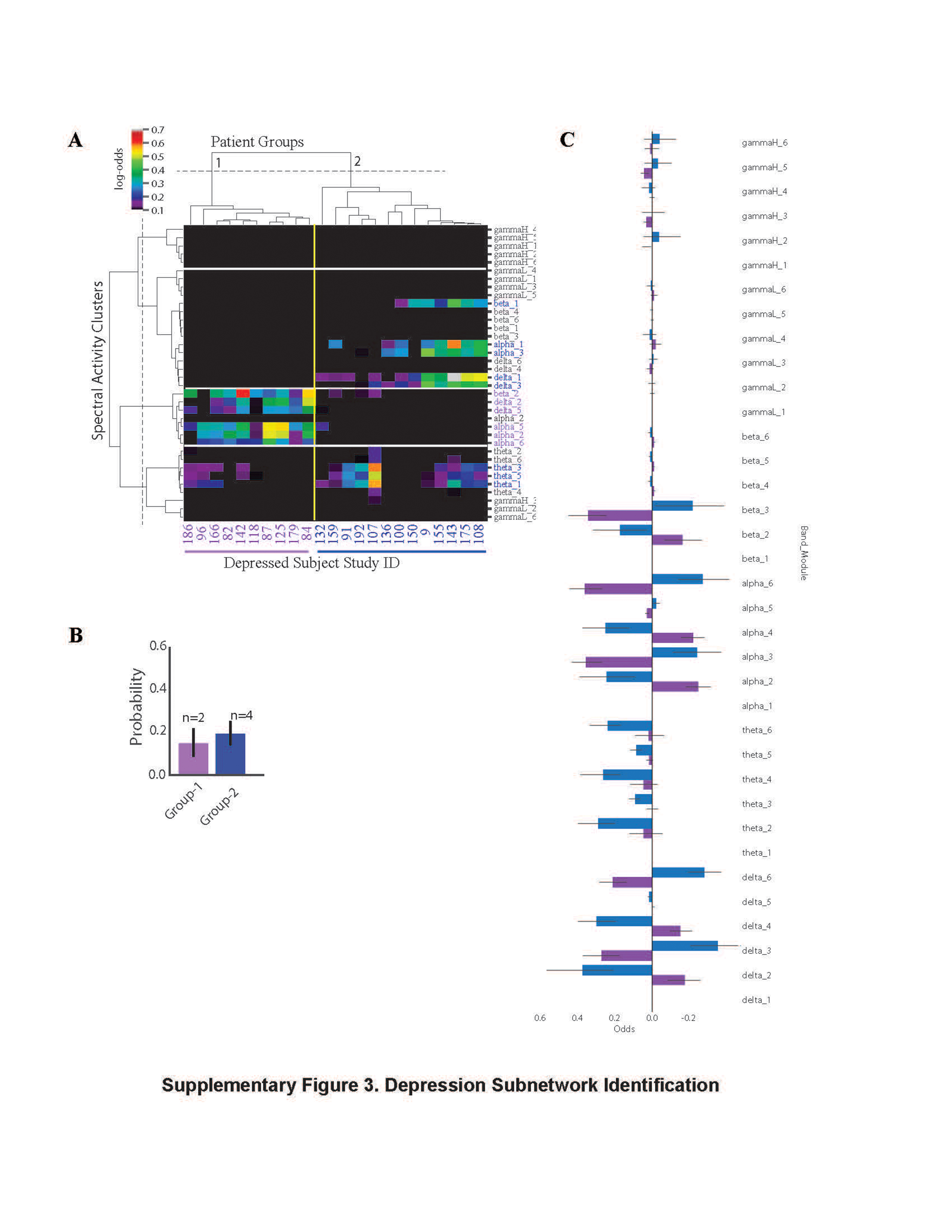

Supplement: Supplementary file 4 [file Image_3.JPEG]
